# Evaluation of consistency in adverse event reporting between trial registry and publications in COVID-19 pharmacological intervention trials

**DOI:** 10.1007/s11096-026-02130-2

**Published:** 2026-04-13

**Authors:** Mia Strikić, Shelly Melissa Pranić

**Affiliations:** 1 Department of Mental Health, Teaching Institute for Public Health Split, Vukovarska 46, 21000 Split, Croatia; 2 Department of Public Health, University of Split School of Medicine, Šoltanska 2A, 21000 Split, Croatia; 3 Cochrane Croatia, Šoltanska 2A, 21000 Split, Croatia

**Keywords:** Adverse events, ClinicalTrials.gov, COVID-19, Drug safety, Randomized controlled trials

## Abstract

**Introduction:**

Complete and consistent reporting of adverse events (AE) affects decisions in clinical practice. We conducted a cross-sectional study aiming to assess transparency and completeness of reported adverse events from randomized clinical trials (RCTs) on pharmacological interventions, including biologicals, to treat Coronavirus disease 2019 (COVID-19) registered on ClinicalTrials.gov on or after January 1, 2020, and updated on or before May 31, 2021, along with corresponding publications.

**Aim:**

We aimed to assess the completeness and consistency of adverse event and all-cause mortality reporting between ClinicalTrials.gov and corresponding publications.

**Method:**

We conducted a cross-sectional comparison of safety reporting between ClinicalTrials.gov registry and peer-reviewed publications of RCTs investigating COVID-19 pharmacological interventions, including biologicals. Two authors evaluated RCTs to reach κ ≥ 0.80.

**Results:**

A total of 68 trials were assessed for discrepancies in adverse event and all-cause mortality data. Thirty-one (46%) were industry-funded, and 44 (65%) were double-blind randomized clinical trials. Forty-nine (72%) publications had discordant counts of serious adverse events (SAE) descriptions, 35 (51%) had discrepancies in the number of patients affected by SAE, and 11 (16%) omitted all-cause mortality compared to the records in the ClinicalTrials.gov registry.

**Conclusion:**

Discrepant reporting of AEs and essential trial data was high in trials on COVID-19 therapeutics.

**Supplementary Information:**

The online version contains supplementary material available at 10.1007/s11096-026-02130-2.

## Impact statements

- Adverse event and mortality data for COVID-19 pharmacological interventions are frequently reported inconsistently between ClinicalTrials.gov and corresponding journal publications.
- Reliable and consistent safety data reporting is essential for informed medication review, patient counselling, and clinical decision-making in pharmacy practice.
- Strengthening adherence to reporting standards could improve decision- making and patient safety.

## Introduction

In this study, an adverse event was defined as any unexpected or expected, and undesirable, event associated with a drug [1–3] and potentially causes harmful economic and health consequences [4]. Reporting adverse events is essential to patient safety and is a legal requirement in the US for certain trials [5–7]. In the context of the past COVID-19 pandemic, the use of repurposed drugs and biologicals for the treatment of COVID-19 carried risks of adverse events such as death [8–11]. This study focuses on pharmacological interventions, including biologicals evaluated for COVID-19 treatment, rather than preventive interventions. Administration of COVID-19 therapeutics remains important even in the face of available COVID-19 vaccines because of inequitable access or ineligibility for vaccination, as well as the emergence of Severe Acute Respiratory Syndrome Coronavirus 2 (SARS-CoV-2) variants [8, 12–14]. In the context of COVID-19 pharmacological trials, where rapid evidence generation was essential, inconsistencies in adverse event reporting may have implications that extend beyond individual trial interpretation, affecting downstream analyses, systematic reviews, and automated safety surveillance [15, 16]. Recent findings on electronic health records show that inconsistencies in adverse event reporting may arise from legitimate methodological or reporting differences [17]. Moreover, some studies have shown that the interpretability and reliability of derived clinical insights depend primarily on the consistency, completeness, and contextual clarity of the underlying data rather than on analytical sophistication alone [18, 19].

Public clinical trial registries were designed to promote transparent and structured clinical trial data. Registry records often represent complete safety information and serve as an important reference for trial results. Therefore, we conducted the first study to assess the completeness and consistency of safety data on pharmacological interventions to treat COVID-19 in ClinicalTrials.gov registry and corresponding publications in peer-reviewed journals [20].

Thus, in this study we aimed to assess the completeness and consistency of adverse event and all-cause mortality reporting between ClinicalTrials.gov and corresponding publications.

## Aim

We aimed to assess the completeness and consistency of adverse event and all-cause mortality reporting between ClinicalTrials.gov and corresponding publications.

## Method

In this cross-sectional study, we followed the Strengthening the Reporting of Observational Studies in Epidemiology (STROBE) reporting guidelines (Additional File 1) [21]. The study was registered in the Open Science Framework (OSF) platform (10.17605/OSF.IO/T8NY9).

### Study period and data sources

**Fig. 1:**
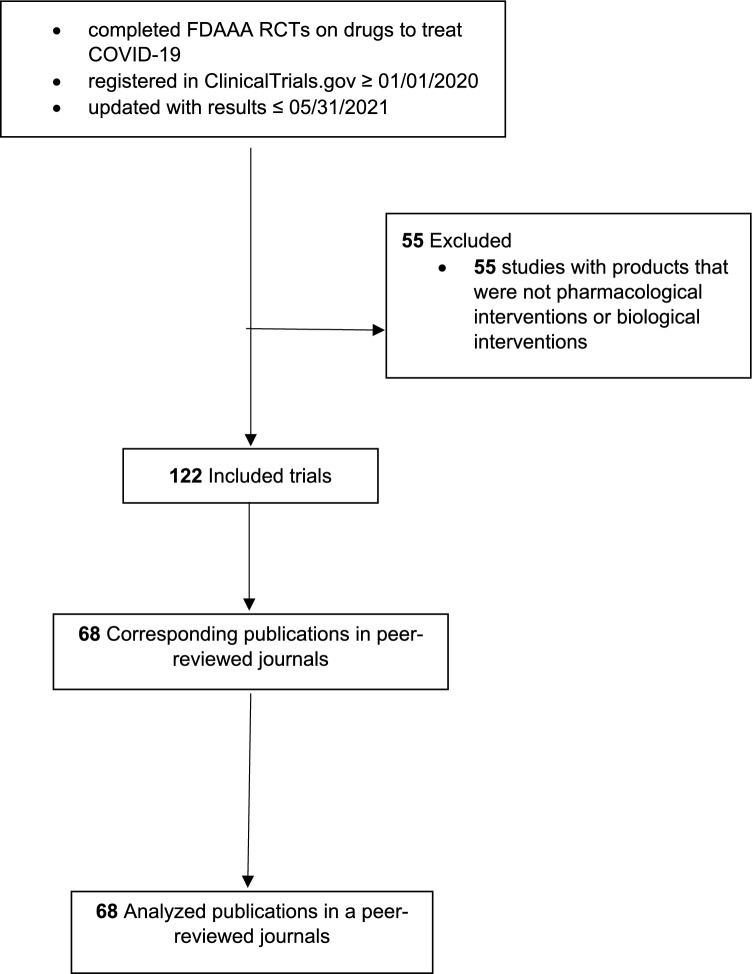
Study flow diagram of trial identification, eligibility assessment, and inclusion, with reasons for exclusion at each stage

We conducted a cross-sectional study on RCTs on COVID-19 pharmacological interventions, including biologicals, comprising RCTs registered on the ClinicalTrials.gov registry. We searched ClinicalTrials.gov using the terms *COVID-19, SARS-CoV-2, coronavirus, pharmacological interventions,* and *biologicals* for interventional trials with results for RCTs registered on or after January 1, 2020, and updated on or before May 31, 2021. We chose this time frame to assess the completeness and integrity of AE reporting due to the rapid development and research of COVID-19 pharmacological interventions during the earliest phase of COVID-19 pandemic. The inclusion criteria were Food and Drug Administration Act (FDAAA) trials, including interventional trials of all recruitment statuses with results, and involving pharmacological and biological interventions to treat COVID-19 disease with the corresponding publications in peer-reviewed journals. The inclusion criteria were not restricted to just RCTs rather clinical trials in registry since other types of clinical trials are registered in the Clinical Trials.gov. We searched PubMed, Web of Science, Google Scholar, and Scopus with the study title and National Clinical Trial (NCT) identifier for corresponding full-text publications in peer-reviewed journals, regardless of whether they were in International Committee of Medical Journal Editors (ICMJE) member journals (Supplementary Material). We excluded trials that studied products that were not pharmacological interventions or biologicals, non-RCT studies (such as observational studies and cohort studies), protocols and non-results trials, editorials and reviews, and preprints (Fig. 1). Publication dates were determined using online publication dates for articles made available ahead of printing [22]. The investigation included trials initiated after the first reported case to the World Health Organization (WHO), with a search period that allowed at least one year for results submission and publication.

### Data extraction and comparisons

Data were extracted from the ClinicalTrials.gov record version available prior to, or contemporaneous with, the first online publication of the corresponding publication in a peer-reviewed journal. Extracted ClinicalTrials.gov adverse events and results data from the registry between the last registration and publication was extracted by one investigator (MS) and verified by another investigator (SMP) [6, 7]. The extracted data was entered into standardized Excel spreadsheet to ensure consistent interpretation of data.

Complete reporting in the registry included tables showed the number of affected participants out of those at risk for each SAE and OAE, as mandatory [23].

In corresponding publications in peer-reviewed journals, complete reporting was considered if there was an explicit statement regarding the occurrence of SAEs, other adverse events (OAEs), or deaths [24]. Following the Consolidated Standards of Reporting Trials (CONSORT) extension, we agreed to use the term “Adverse Event” as defined in ClinicalTrials.gov [24]. The extension was developed to improve quality in randomized controlled trials reporting introduced by the CONSORT 2010 statement. We qualified inconsistent reporting of AEs if there were changes in descriptions, the number of affected participants, or the total AEs between ClinicalTrials.gov and publications.

Adverse events were classified into *serious* and *other adverse events,* following ClinicalTrials.gov definitions. References to adverse events, include both serious and other adverse events. The term “*harm”* was used only in the context of reporting standards, and treatment-emergent adverse events (TEAEs) were considered only when explicitly reported as such in the registry or publication.

Differences in adverse event descriptions were assessed depending on the presence and number of adverse event categories. Equivalent medical terms describing the same clinical event were considered concordant and were not classified as discrepant. When adverse events were reported at different levels of detail in the registry and publication (e.g., we grouped in one source but listed separately in the other), we classified this as discrepancies in adverse event descriptions. A discrepancy in the descriptions was recorded when adverse events were reported as different clinical terms, or were aggregated in one source but disaggregated in the other, or when were reported in one source but omitted in the other. Adverse events and all-cause mortality were considered not to have occurred only when explicitly reported as zero. Absence of reporting of adverse events or all-cause mortality was classified as missing data and recorded as omitted.

We defined numerical discrepancies as any difference in reported numbers between the registry and the publication. No threshold for clinical importance was applied, as the primary objective of this study was to assess completeness and consistency of safety reporting.

Inconsistent reporting was recorded when the underlying integer counts of adverse events, all-cause mortality, or affected participants differed between the ClinicalTrials.gov registry and the corresponding publication.

Two investigators (MS and SP) independently extracted the data from the trial data and corresponding publications to minimize potential biases. Disagreements in data extraction were resolved through the discussion until consensus was reached [6, 25]. The reliability of extractions concerning differences in SAEs description was high (kappa 1.00 (95%CI 1.00–1.00) between the registry and corresponding publications. We determined the interrater reliability of the independent assessments of changes in results and adverse events data using Cohen's kappa (κ).

### Statistical analysis

Data was extracted independently by two authors in the Excel spreadsheet and coded prior to analysis. Extracted data were reported descriptively as frequencies (n) and percentages (%) according to their respective categories. The Chi-square test was used to compare frequencies. We reported Cohen’s effect size (ϕ) for Chi-square analysis and (κ) with corresponding 95% confidence intervals (CI). Cohen’s effect sizes ranges small (0.10) to large (0.50), where 0.30 represents a moderate magnitude [26]. We used IBM SPSS Statistics for Windows, version 22.0 (IBM Corp., Armonk, N.Y., USA) for all analyses.

### Ethics approval

There was no need for ethics approval since we did not include any participants or animals in our cross-sectional study.

## Results

### Trial characteristics

We retrieved 177 trials from ClinicalTrials.gov registry that met our inclusion criteria. We selected 122 eligible RCTs after applying our exclusion criteria. Trial baseline characteristics were reported as recorded by investigators in ClinicalTrials.gov. Fifty-five RCTs were excluded due to exploring products that were not pharmacological or biological interventions. Of those 122 RCTs, we found 68 corresponding publications in peer-reviewed journals (Fig. 1).

Most trials with results published (n = 41/68, 60%) did not start before registration. Most trials were double-blind (n = 44/68, 65%), randomized (n = 64/68, 94%), industry-funded (n = 31, 46%), and placebo-controlled (n = 41/68, 60%). Drugs were the most common intervention in the publications (n = 44/68, 65%), primarily in the parallel assignment (n = 59/68, 87%). Biological interventions were used in 34% publications (n = 23/68).

Trial design characteristics, including randomization type, masking, and control type, are reported in Table 1 as entered by investigators in ClinicalTrials.gov.

**Table 1:** Study characteristics of COVID-19 pharmacological interventions RCTs (n = 68) with published results for trials registered on or after January 1, 2020, and updated on or before 31 May 2021, in ClinicalTrials.gov

Baseline characteristics		Reporting rate, n (%)
Phase*	N/A	0 (0)
	1	3 (4)
	1/2	1 (2)
	2	15 (22)
	2/3 3 4 Unspecified	3 (4) 19 (28) 0 (0) 27 (40)
Masking†	Open label	10 (15)
	Single blind	3 (4)
	Double blind	44 (64)
	Triple blind	0 (0)
	Quadruple blind Unspecified	1 (2) 10 (15)
Control	Placebo	41 (60)
	Active	15 (22)
	Both No control group Not receiving anything	8 (12) 3 (4) 1 (2)
Assignment	Single group	2 (3)
	Parallel	59 (87)
	Cross-over	1 (2)
	Sequenced Unspecified	1 (2) 5 (6)
Total		68 (100)

COVID-19, Coronavirus Disease 2019; RCT, Randomized Controlled Trial

*, † Data as entered by investigators in ClinicalTrials.gov

n refers to the number of trials; percentages were calculated using the total number of trials in the table as the denominator

The most commonly used intervention was hydroxychloroquine (n = 8/68, 12%), which was used in combination with azithromycin (n = 1/68, 2%), followed by remdesivir (n = 6/68, 9%), which was used in combination with tocilizumab (n = 1/68, 2%) and interferon beta-1a (n = 1/68, 2%).

Adverse event and death reporting.

Most trials discrepantly reported AEs (Table 2), including SAEs (Table 3), OAEs (Table 4) and all-cause mortality data (Table 5).

**Table 2:** Reporting adverse events for COVID-19 pharmacological interventions RCTs (n = 68) with published results for trials registered on or after January 1, 2020, and updated on or before 31 May 2021 in ClinicalTrials.gov

	ClinicalTrials.gov	Publication	Effect size^a^ (95% CI)	*P*-value^b^
*Reporting rate, n (%)*				
SAEs	67 (99)	57 (84)	0.26 (0.10–0.42)	0.003
OAEs	68 (100)	57 (84)	0.30 (0.14–0.46)	0.001
Deaths	68 (100)	54 (79)	0.34 (0.18–0.50)	0.001
*AEs reported as zero (n, %)*				
SAEs	9 (13)	9 (13)	N/A	1.000
OAEs	9 (13)	3 (4)	N/A	0.070
Deaths	21 (31)	12 (18)	N/A	0.072
AEs omitted (n, %)				
SAEs	1 (2)	11 (16)	0.26 (0.10–0.42)	0.003
OAEs	0 (0)	11 (16)	0.30 (0.14–0.46)	0.001
Deaths	0 (0)	14 (21)	0.34 (0.18–0.50)	0.001

AE, Adverse Event; COVID-19, Coronavirus Disease 2019; CI, Confidence Interval; RCT, Randomized Controlled Trial; SAE, Serious Adverse Event; OAE, Other Adverse Event

n refers to the number of trials; percentages were calculated using the total number of trials in the table as the denominator

^a^Cohen’s effect size (ϕ); ranges from 0.10 to 0.50, where 0.30 represents a moderate magnitude

^b^Chi-square test with the significance level considered at < 0.05

**Table 3:** Reporting serious adverse events (SAEs) for COVID-19 pharmacological interventions RCTs (n = 68) with published results for trials registered on or after January 1, 2020, and updated on or before 31 May 2021 in ClinicalTrials.gov

	Reporting rate, n (%)
*Different number of SAEs descriptions*	
Yes	49 (72)
No	19 (28)
*If a different number of SAEs descriptions (n = 49)*	
Less in the register	6 (12)
More in the register	33 (67)
Omitted in the publication (n = 68)	11 (16)
Not applicable (N/A) (n = 68)	19 (28)
*Different sum of affected participants by SAEs*	
Yes	35 (51)
No	33 (49)
*If a different sum of affected participants (n = 35)*	
Less participants in the register	8 (23)
More patients in the register	17 (49)
Omitted sum of participants in the publication (n = 68)	11 (16)
Not applicable (N/A) (n = 68)	33 (49)

AE, Adverse Event; COVID-19, Coronavirus Disease 2019; RCT, Randomized Controlled Trial; SAE, Serious Adverse Event; OAE, Other Adverse Event

n refers to the number of trials; percentages were calculated using the total number of trials in the table as the denominator

**Table 4:** Reporting other adverse events (OAEs) for COVID-19 pharmacological interventions RCTs (n = 68) with published results for trials registered on or after January 1, 2020, and updated on or before 31 May 2021 in ClinicalTrials.gov

	Reporting rate, n (%)
*Different number of OAEs descriptions*	
Yes	55 (81)
No	13 (19)
*If a different number of OAEs descriptions (n = 55)*	
Less OAEs descriptions in the register	23 (42)
More OAEs descriptions in the register	21 (38)
Omitted description of OAEs in the publication (n = 68)	11 (16)
Not applicable (N/A) (n = 68)	13 (19)
*Different sum of affected patients by OAEs*	
Yes	55 (81)
No	13 (19)
*If a different sum of affected patients by OAEs (n = 55)*	
Less participants affected by OAEs in the register	30 (55)
More participants affected by OAEs in the register	14 (25)
Omitted number of affected participants by OAEs in the publication (n = 68)	11 (16)

COVID-19, Coronavirus Disease 19; RCT, Randomized Controlled Trial; OAE, Other Adverse Events

n refers to the number of trials; percentages were calculated using the total number of trials in the table as the denominator

**Table 5:** All-cause mortality reporting in COVID-19 pharmacological interventions RCTs (n = 68) with published results for trials registered on or after January 1, 2020, and updated on or before 31 May 2021 in ClinicalTrials.gov

	ClinicalTrials.gov, n (%)	Publication, n (%)	Effect size (95% CI)^a^	*P*-value^b^
Total number of trials reporting on all-cause mortality	68 (100)	54 (79)	0.34 (0.18–0.50)	0.001
Total number of trials omitting all-cause mortality	0 (0)	14 (21)	0.34 (0.18–0.50)	0.001
Total number of trials reporting on all-cause mortality as zero	21 (31)	12 (18)	N/A	0.072

CI, confidence interval; COVID-19, Coronavirus Disease 19; RCT, Randomized Controlled Trial

n refers to the number of trials; percentages were calculated using the total number of trials in the table as the denominator

^a^Cohen’s effect size (ϕ); ranges from 0.10 to 0.50, where 0.30 represents a moderate magnitude

^b^Chi-square test with the significance level considered at < 0.05

All trials in ClinicalTrials.gov reported OAEs and deaths and only one trial did not report SAEs (Table 2). The frequency threshold for OAEs was reported in all trials in the registry and 24% trials in the publication. Reporting of and omission of SAEs between ClinicalTrials.gov and publications was significantly different with a small to moderate effect size (Table 2). The difference in reporting rate and omission of OAEs and deaths was moderate (Table 2.)

Discrepancies in the number of SAE descriptions were recorded in 72% trials, with the results published. Of those 49 trials, 67% had a larger number of SAE descriptions in the ClinicalTrials.gov registry and only 12% had a smaller number of SAEs descriptions in the ClinicalTrials.gov registry than in the corresponding publications. We recorded that SAEs descriptions were omitted in 11 trials, and for 18 trials we recorded as “Not applicable” as there were no values reported in either one of the sources which precluded assessing differences in the number of SAE descriptions between the registry and corresponding publications (Table 3). Predominantly, less participants were affected by SAEs in the registry than in articles.

Difference in the number of OAEs descriptions between ClinicalTrials.gov registry and the publications was recorded in the majority of reported trials with the most dominant change as less descriptions in the registry. In 13 trials, no differences in OAE descriptions were identified between the registry and the publication (Table 4).

A total of 14 trials with published results did not report deaths. In the registry, 21 trials reported zero deaths, where only 12 trials reported zero deaths in the corresponding publications, indicating discordance between the two sources (Table 5). The differences in the total number of trials reporting on and omitting all-cause mortality were significant and showed moderate effect sizes (Table 5).

## Discussion

To our knowledge, this is the first study to evaluate the concordance of adverse events reporting from COVID-19 RCTs in ClinicalTrials.gov and corresponding publications in peer-reviewed journals. Our cross-sectional study assessed discrepancies in adverse events and all-cause mortality reporting from RCTs on pharmacological interventions to treat COVID-19 and presented inconsistent and incomplete reporting in corresponding publications. We found that AE data discrepancies were high between the registry and publications. Analyses were restricted to registry versions available at or before publication date to minimize potential bias from post-publication changes related to modifications of registry data. Our study showed that discrepancies in reporting SAEs occurred in 72% of publications and for OAEs in 81% of publications. Discrepancies in reporting the death of participants occurred in 59%. Moreover, the differences between sources showed small-to-moderate effect sizes, suggesting that discrepancies were not negligible and may have implications for the interpretation of safety data.

More than a half of the analyzed publications reported SAEs, and it is evident that publications underreported SAEs in relation to the ClinicalTrials.gov registry, indicating a pattern of relative underreporting in journal articles. This may lead to underestimation of treatment related risks in clinical practice. Almost a quarter of the publications explicitly reported zero serious adverse events. As a result, complete omission of serious adverse event reporting was less frequent, since some publications provided an explicit statement indicating that no events occurred. Some authors found similar discrepancies in the SAEs reporting and reinforced concerns regarding consistency and completeness of safety reporting [27–33].

Only 16% of the publications omitted OAE reporting. We observed discrepancies in OAE reporting with more OAE descriptions reported in ClinicalTrials.gov registry than in the corresponding publications in more than half of the trials. Discrepancies in the reporting of OAEs were described by some authors [34, 35], but most authors have studied discrepancies in SAEs reporting. Complete and consistent reporting of adverse events contributes to better decision-making and patient safety through reducing the underestimation of adverse events from treatments.

Some discrepancies between ClinicalTrials.gov and corresponding publications may reflect legitimate differences in reporting practices. The legitimate reasons may include variations in adverse event classification, journal requirements, and different reporting standards between journals and registries. On the other hand, discrepancies may occur due to omission of reporting SAEs, OAEs and/or death data.

Discrepancies in death reporting were found in more than half of the corresponding publications, from which only 18% of publications reported the all-cause mortality as zero. Our study also shows that the frequency of the reporting of deaths between the two sources was discordant. Deaths are variably reported in both sources, and some authors described inconsistent death reporting in their studies [32, 36, 37]. The correct reporting of all-cause mortality is not only methodologically important, but also ethically essential. Inconsistent and incomplete mortality reporting may reduce transparency and limit the ability of clinicians and policymakers to fully assess the safety of pharmacological interventions. It may also complicate trial oversight processes, including safety monitoring and data review processes.

Recent findings in natural language processing highlight promising opportunities to improve transparency in adverse event reporting. Studies have demonstrated the ability to identify underreported or inconsistently reported events with greater sensitivity than traditional structured data sources [16–18]. These findings suggest that reliance on manually curated registry entries may contribute to discrepancies similar to those observed in the present study.

A problem with adverse event overreporting was noted in 2021 during mass immunization with COVID-19 vaccines [38]. Along with our findings these observations highlight challenges in ensuring complete and consistent safety reporting across different data sources. Research on discrepancies in adverse events reporting has stressed that there is a need for improvement and application of regulatory requirements by trialists [34, 39]. Suggestions for improved transparent adverse events reporting include complete reporting of essential data elements in clinical trial registries before a trial is considered fully registered [34]. Further, an author-generated checklist was suggested to help explain discrepancies in data to editors for articles submitted to journals [40].

The conducted study has several strengths. We used a systematic approach to assess adverse event and mortality reporting between two sources. Data extraction was performed independently by two investigators, with potential disagreements resolved through consensus, in order to minimize individual bias. The focus on clinical trials together with randomized controlled trials on pharmacological interventions, including biologicals to treat COVID-19 disease, provides timely insight into safety reporting during a period of rapid evidence dissemination.

There are several limitations to this study. Firstly, our selection of trials was restricted to trials registered in ClinicalTrials.gov registry and does not capture COVID-19 trials registered in other registries. Findings showed that the majority of COVID-19 clinical trials have been reported to be registered in the Chinese Clinical Trials Registry [41], so trials registered outside ClinicalTrials.gov were not included, which limits the representativeness of the study population. Moreover, the interpretation of discrepancies and completeness relied on criteria applied to registry and publications. However, the reliability of the extractions concerning discrepancies between the latest registered data and data in publications for the two raters was high. Also, we searched ClinicalTrials.gov registry and corresponding publications in bibliographic database prior to registering study on OSF platform, so this is a limitation. Although the methodology was not changed after data extraction, retrospective registration may be considered less rigorous than prospective registration. In addition, preprints were excluded from our analysis, reflecting publication delays, selective publication, or non-publication of trial results. Finally, discrepancies between registry and corresponding publications were not identified by severity, and associations between trial characteristics and discrepancies were not assessed, limiting clinical significance.

Discrepancies in the reporting of adverse events from COVID-19 trials diminish the transparency of the safety profile of essential COVID-19 treatments. There is a need to improve adverse event and mortality data reporting between ClinicalTrials.gov and corresponding publications through policy recommendations in order to upgrade decision making in treatments for COVID-19 disease. At the journal level, requiring cross-checking of reported safety data between registries and manuscripts could help in early identification of inconsistencies and reduce incomplete reporting while submitting the manuscript. Moreover, targeted educational interventions- such as training on CONSORT Harms guidelines, structured adverse event reporting in clinical trial registries, and regulatory requirements for safety data submission, could improve the reliability of reported safety data. These measures could support more accurate risk–benefit assessment and improve the transparency of clinical trial data for clinicians, researchers, and policymakers.

## Conclusion

In our novel cross-sectional study, we found high incidence of discordant reporting between two sources for AEs, including all-cause mortality data. Continuous research interest-holder involvement to reduce discrepant reporting from COVID-19 RCTs can support transparent and informed reporting of adverse events for patient safety and clinicians’ treatment decisions.

## Supplementary Information

Below is the link to the electronic supplementary material.

Supplementary file1 (DOCX 14 KB)

Supplementary file2 (DOCX 14 KB)

## Data Availability

The datasets generated and analysed during the current study are available in the Open Science Framework repository, doi: [10.17605/OSF.IO/T8NY9] (https:/doi.org/10.17605/OSF.IO/T8NY9).
