# Supplementary material for: Evaluation of consistency in adverse event reporting between trial registry and publications in COVID-19 pharmacological intervention trials
Source: Int J Clin Pharm. 2026 Apr 13;48(4):1510–8. doi: 10.1007/s11096-026-02130-2 (PMC13369681; doi:10.1007/s11096-026-02130-2)
Supplement: Supplementary file 1 — Supplementary file1 (DOCX 14 KB) [file 11096_2026_2130_MOESM1_ESM.docx]

Supplementary Material 1.

Table S1. Coding manual for classification of reporting discrepancies

| **Discrepancy category** | **Operational definition** | **Classification rule** | **Illustrative example** |
| --- | --- | --- | --- |
| **Terminology (concordant)** | Use of synonymous or clinically equivalent terms describing the same adverse event | Classified as **concordant** (not a discrepancy) | “Myocardial infarction” vs. “heart attack” |
| **Different AE descriptions** | Difference in the number or type of distinct adverse event categories reported between sources | Classified as **discrepant** | SAE reported in ClinicalTrials.gov but not mentioned in the publication |
| **Aggregation vs. granularity** | Adverse events reported as grouped categories in one source and as multiple specific events in the other | Classified as **discrepant** due to differences in number and specificity of categories | “Cardiac disorders” vs. arrhythmia, myocardial infarction |
| **Numerical discrepancy** | Any difference in underlying integer counts of adverse events or affected participants | Classified as **discrepant**, regardless of magnitude | 5 SAEs vs. 7 SAEs |
| **Rounding / percentage differences** | Differences attributable solely to rounding, denominator choice, or percentage presentation | Classified as **concordant** (not a discrepancy) | 3/100 (3.0%) vs. 3/99 (3.0%) |
| **Zero events** | Explicit reporting that no events occurred | Classified as **zero events** | “No deaths reported” |
| **Missing data (omitted)** | Absence of reporting of adverse events or mortality | Classified as **missing data / omitted**, not as zero events | AE section not reported in the publication |
