# Supplementary material for: Evaluation of consistency in adverse event reporting between trial registry and publications in COVID-19 pharmacological intervention trials
Source: Int J Clin Pharm. 2026 Apr 13;48(4):1510–8. doi: 10.1007/s11096-026-02130-2 (PMC13369681; doi:10.1007/s11096-026-02130-2)
Supplement: Supplementary file 2 — Supplementary file2 (DOCX 14 KB) [file 11096_2026_2130_MOESM2_ESM.docx]

Supplementary Note 1. Search strategy for ClinicalTrials.gov (RCTs registered from January 1, 2020, and updated on May 31, 2021)

Search strategy in the Advanced Search feature:

terms: *COVID-19, SARS-CoV-2, coronavirus, pharmacological interventions, biologicals*

study phase: all study phases

study type: interventional type

study results: with results

Supplementary Note 2. Search strategy for publications in PubMed, Scopus, Google Scholar, Web of Science using the study title and NCT identifier number from January 1, 2020, and updated on May 31, 2021

Search strategy for PubMed: ("Study title") OR (NCT number)

Search strategy for Scopus: ("Study title") AND (NCT number)

Search strategy for Google Scholar: ("Study title") AND (NCT number)

Search strategy for Web of Science: ("Study title") AND (NCT number)
